# Prospective Application of Nanoencapsulated *Bacillus amyloliquefaciens* on Broiler Chickens’ Performance and Gut Health with Efficacy against *Campylobacter jejuni* Colonization

**DOI:** 10.3390/ani13050775

**Published:** 2023-02-21

**Authors:** Hesham Ismail, Doaa Ibrahim, Shorouk El Sayed, Ali Wahdan, Reham M. El-Tarabili, Waleed Rizk El-Ghareeb, Bassam Abdullah Alhawas, Badr Abdul-Hakim Y. Alahmad, Sherief M. Abdel-Raheem, Marwa I. Abd El-Hamid

**Affiliations:** 1 Department of Public Health, College of Veterinary Medicine, King Faisal University, P.O. Box 400, Hofuf 31982, Al-Ahsa, Saudi Arabia; 2 Food Hygiene Department, Faculty of Veterinary Medicine, Assiut University, Assiut 71526, Egypt; 3 Department of Nutrition and Clinical Nutrition, Faculty of Veterinary Medicine, Zagazig University, Zagazig 44511, Egypt; 4 Department of Microbiology, Faculty of Veterinary Medicine, Zagazig University, Zagazig 44511, Egypt; 5 Department of Bacteriology, Immunology and Mycology, Faculty of Veterinary Medicine, Suez Canal University, Ismailia 41522, Egypt; 6 Food Control Department, Faculty of Veterinary Medicine, Zagazig University, Zagazig 44519, Egypt; 7 Department of Animal Nutrition and Clinical Nutrition, Faculty of Veterinary Medicine, Assiut University, Assiut 71526, Egypt

**Keywords:** probiotic-loaded nanoparticle, performance, *C. jejuni*, colonization, barrier function, poultry

## Abstract

**Simple Summary:**

The emergence of antibiotics resistance is a warning sign to limit antibiotics usage as growth promoters in the poultry industry. Probiotics served as superior candidates for replacing antibiotics in the poultry sector. However, the beneficial functions of probiotics did not reach their targeted outcomes owing to the harsh environment in the poultry gut. In this context, evolution of biotechnological aids offers new avenues for increasing bioavailability and beneficial efficacy of in-feed additives including probiotics. Therefore, encapsulation of *Bacillus amyloliquefaciens* (*B. amyloliquefaciens*) into nanocarriers boosted its growth-promoting purposes and consequently modulated the functions of digestive enzymes and kept the microbiota homeostasis towards the beneficial ones. The strengthening capability of *B. amyloliquefaciens*-loaded nanoparticles for broilers’ gut barrier limited *Campylobacter jejuni* (*C. jejuni*) colonization and shedding. This superior outcome would in turn interrupt the transmission cycle of *C. jejuni* through the food chain and consequently protect against its adverse consequences in humans.

**Abstract:**

Probiotics as novel antibiotics’ substitutes are verified to provide barriers for hindering the colonization of enteric bacterial pathogens with nutritional benefits. For enhancement of the probiotics’ effectiveness, their integration within nanomaterials is a paramount tool to support the progress of new compounds with functional features. Therefore, we addressed the impact of effective delivery of probiotics (*Bacillus amyloliquefaciens*) loaded nanoparticles (BNPs) on performance and *Campylobacter jejuni* (*C. jejuni*) shedding and colonization in poultry. Two hundred Ross broiler chickens were divided into four groups fed various BNP levels: BNPs I, BNPs II, BNPs III, and BNPs-free diets for 35 days. Nanoparticles delivery of probiotics within broiler diets improved growth performance as reflected by higher body weight gain and superior feed conversion ratio, especially in BNPs II- and BNPs III-fed groups. In parallel, the mRNA expression levels of digestive enzymes encoding genes (*AMY2a*, *PNLIP*, *CELA1*, and *CCK*) achieved their peaks in BNPs III-fed group (1.69, 1.49, 1.33, and 1.29-fold change, respectively) versus the control one. Notably, with increasing the levels of BNPs, the abundance of beneficial microbiota, such as *Bifidobacterium* and *Lactobacillus* species, was favored over harmful ones, including *Clostridium* species and *Enterobacteriaceae*. Birds fed higher levels of BNPs displayed significant improvement in the expression of barrier functions-linked genes including *DEFB1*, *FABP-2*, and *MUC-2* alongside substantial reduction in cecal colonization and fecal shedding of *C. jejuni*. From the aforementioned positive effects of BNPs, we concluded their potential roles as growth promoters and effective preventive aids for *C. jejuni* infection in poultry.

## 1. Introduction

Feed additives as growth promoters have a robust impact on the production cost of broilers for covering alterations in profits due to instability in feed costs [1,2]. The extensive use of antibiotics as feed additives for enhancing poultry growth performance and treatment of bacterial infections leads to the emergence of multidrug resistant (MDR) bacterial strains [3,4,5,6,7,8]. Up until now, research is in progress exploring feed additives that can substitute antibiotics as prophylactics and growth promoters in poultry production. Probiotics became prospective antibiotic alternative additives in broilers owing to their impact on growth performance and the well-being of broilers compared to antibiotics [9,10,11]. The main favorable effects of probiotics correlate largely with improved feed bioavailability and digestibility, boosting the immune system, saving health, and providing superior carcass composition [12,13]. They have immunomodulatory roles in enhancing immunity against microbes and preventing exaggerated inflammatory responses, and they serve as biological barriers to protect the epithelial cells from being breached by pathogenic bacteria and to maintain epithelial integrity [14]. As well, probiotic bacteria are able to impede the growth of pathogenic microflora in the gastrointestinal tract of birds owing to their roles in nutrient depletion, blockage of pathogens’ target receptors on epithelial cells, and the creation of natural antibacterial products known as bacteriocins [15,16,17,18]. In addition, the early institution of probiotics in the gut can provide a barrier against foodborne pathogen colonization [19]. Bacillus-based probiotics have unique properties comprising growth promotion, immunomodulation, competitive exclusion [20,21], and production of a variety of extracellular substances and antimicrobial peptides against a wide range of pathogens [22,23]. *Bacillus amyloliquefaciens* (*B. amyloliquefaciens*) is a strong *Bacillus* species that produces numerous extracellular enzymes such as cellulase, metalloproteases, proteases, and α-amylases that augment digestibility and nutrient absorption in addition to overall gut immune functions [24,25]. Moreover, *B. amyloliquefaciens* produces bacteriocins [26] with a consequence of gut resistance to infection, and it reduces noxious gases emission in chickens [27]. Additionally, *B. amyloliquefaciens* CECT 5940 possesses a wide range of antimicrobial activity that can ultimately improve broilers’ health [28].

Disruption in the elegant interaction between gut microbiota, intestinal epithelium barrier, and host immunity plays a crucial role in the development of acute bacterial enteritis including campylobacteriosis [29,30], which is caused by *Campylobacter jejuni* (*C. jejuni*) and is considered one of the most serious foodborne pathogens causing zoonotic bacterial gastroenteritis in humans. Poultry are a major reservoir for *C. jejuni* as they provide it with an optimal temperature (42 °C) in their gastrointestinal tract, which is required for its colonization and proliferation [19,31]. *Campylobacter jejuni* inhabits avian cecum with approximately 10^6^–10^8^ colony forming unit (CFU)/g without clinical illness [32]. Pondering this situation, exploring ongoing promising strategies to reduce *C. jejuni* colonization in poultry and therefore in humans is needed. Administration of beneficial live probiotics can rescue the intestinal microbiota ecosystem balance, inhibit exaggerated immune responses against innocuous antigens, and hinder pathogenic bacterial colonization by competitive exclusion [33]. Recently, *B. amyloliquefaciens* CECT 5940 could probably improve growth rate, nutrient intake, gut function, and negative impacts of necrotic enteritis challenge [34]. However, its usage as an alternative to antibiotics is still limited because a harsh gut environment of gastric acids and bile salts may kill it and decrease its bioavailability with a consequent demand for high doses and long-term use of probiotics to exert their curative functions [35,36,37,38]. Therefore, to overcome these limitations and use the advantages of available advancement in technology, recent studies have investigated different formulation methods for effective oral delivery of probiotics to protect them from harsh gut environments and sustain their therapeutic effects [35,39]. Nanoparticle formulations provide a new gateway for achieving a safe delivery system for the ingredients used in feed by increasing their potency and concentration at their target sites [36,40,41]. Moreover, using natural biodegradable biopolymers for encapsulation of these ingredients is widely applied [42]. Based on this technology, co-encapsulation of probiotics strains for their engineering to improve their therapeutic efficiency has been reported [43]. However, the effect of using *B. amyloliquefaciens*-loaded nanoparticles (BNPs) as alternatives to antibiotics for growth promotion and competing against campylobacteriosis needs to be defined. Therefore, we investigated for the first time in this study the effect of feeding poultry with BNPs on growth performance, barrier function, and immunity of broiler chickens in addition to its efficiency on cecal colonization of *C. jejuni* and its shedding in poultry excreta.

## 2. Materials and Methods

### 2.1. Ethical Statement

All experimental procedures, bird rearing, and management were conducted upon approval from animal resources at the Faculty of Veterinary Medicine, Zagazig University, Egypt according to the rules of the Institutional Animal Care and Use Committee (ZU-IACUC/2/F/337/2022 approval number).

### 2.2. Bacillus amyloliquefaciens-Loaded Nanoparticles (BNPs)

**Figure 1:**
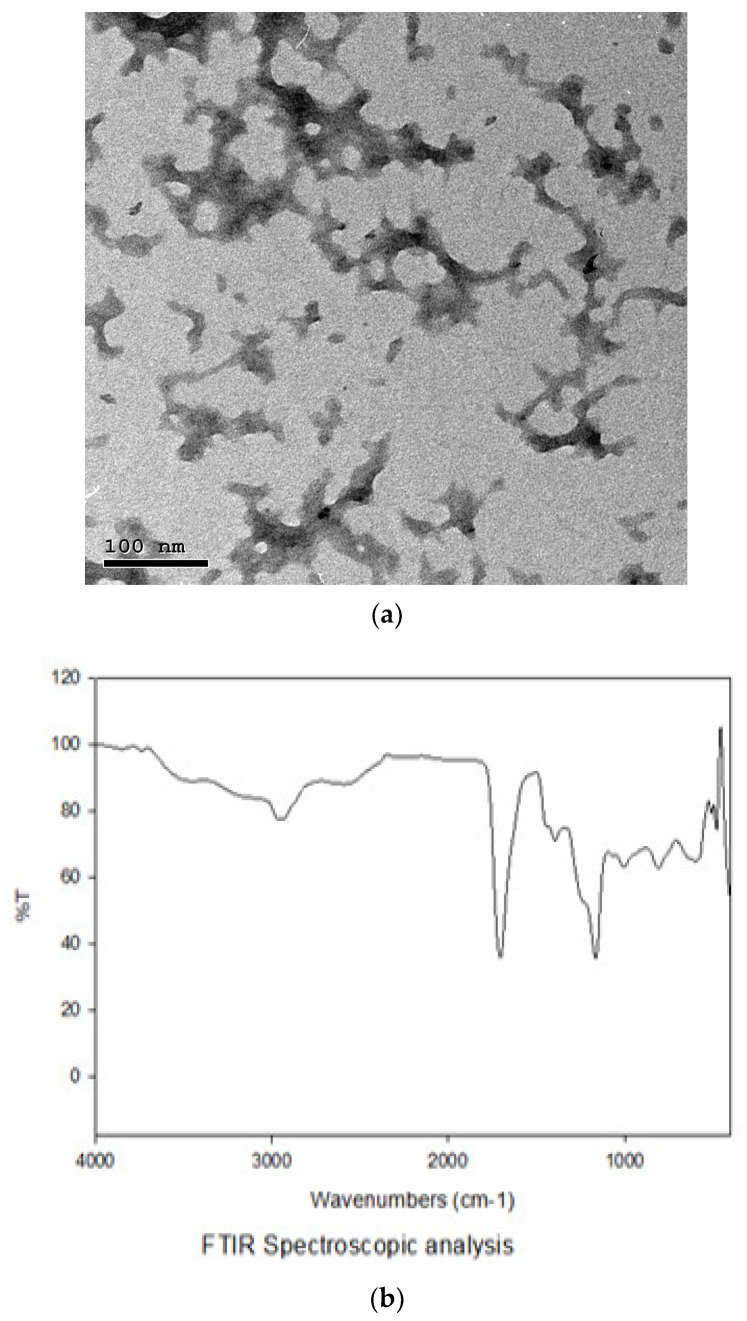
Characterization of Bacillus amyloliquefaciens-encapsulated nanoparticles using transmission electron microscopy (**a**) and Fourier-transform infrared spectroscopy (**b**).

The strain of *B. amyloliquefaciens* probiotic CECT 5940 obtained from Evonik Nutrition and Care GmbH was propagated in Luria–Bertani (Oxoid, Hampshire, UK) broth and incubated at 37 °C. Then, *B. amyloliquefaciens* was stored in the bacterial cryopreservation fluid at −80 °C for further experiments. The BNPs were prepared through incorporation of the bacteria into chitosan (0.4%, *w*/*v*) (Sigma-Aldrich, St. Louis, MO, USA) nanoparticles as previously described [44]. The prepared BNPs were stored by freezing at −20 °C in a cryoprotectant agent and were then dried and condensed for 18 h at −40 °C using LyoBeta 25™ freeze-dryer (Telstar, Terrassa, Spain). Finally, the dried cells were kept at 4 °C to be protected from light. The nano size and shape of the formulated BNPs were confirmed using transmission electron microscopy and Fourier-transform infrared spectroscopy characterization (Figure 1). 

### 2.3. Birds and Experimental Design Considerations

Two hundred Ross 308 male broiler chickens (42.21 g average initial weight) at 1 day old were provided from a commercial hatchery (Dakahlia Poultry Company, Dakahlia, Egypt). The birds were housed in divided floor pens for separation between groups at the Animal Care Unit at the Faculty of Veterinary Medicine, Zagazig University. The birds were divided randomly into 4 groups; each group had 5 replicates (10 birds each). The first group defined as control was fed the conventional diet and the three other groups were offered the conventional diet supplemented with *B. amyloliquefaciens*-loaded nanoparticles (BNPs) with three different doses [BNPs I (2.5 × 10^5^ CFU/g of feed), BNPs II (5 × 10^5^ CFU/g of feed) and BNPs III (7.5 × 10^5^ CFU/g of feed)]. Both control and tested groups were fed the conventional or BNPs-containing diets for 35 days during the experimental period. All chicks in control and tested groups were co-housed at the same rearing conditions: temperature (33 ± 1 °C), which was gradually decreased every week until it reached 24 ± 2 °C at the end of the experimental period and humidity (around 60%) was constant during the whole experimental period. The diet was formulated according to nutrition specification of a Ross broiler handbook [45] as presented in Table 1. Chemical analyses of all feed ingredients were performed using the standard method as endorsed by the Association of Official Analytical Chemists, AOAC77 [46]. All animals were provided with ad libitum access to water and food during the whole experimental period.

### 2.4. Growth Performance and Mortality Indicators

Individual birds were weighed and feed residues were determined to calculate feed intake for evaluation of different growth parameters of broiler chickens within the growth phases (1–35 days) including body weight gain for each phase [final body weight (g/bird) − initial body weight (g/bird)] and feed conversion ratio (FCR) [feed intake (g/bird)/weight gain (g/bird)] [18,47]. Moreover, mortality rate was recorded during the period from 1 to 35 days.

### 2.5. Sampling Procedure

At the end of the experimental period, the birds (5 per replicate) were weighed and euthanized by cervical dislocation. The intestinal contents and cecal and fecal samples were then aseptically placed in sterile Eppendorf tubes and kept at −80 °C for further quantification of intestinal microbial populations and evaluation of *C. jejuni* colonization and shedding via real-time (quantitative) polymerase chain reaction (qPCR) strategies. Moreover, pancreatic and jejunal samples (around 1 cm each) were excised, flushed three times with phosphate buffered saline, and subjected later to genes expression analysis utilizing reverse transcription-quantitative polymerase chain reaction (RT-qPCR) assays.

### 2.6. Reverse Transcription Quantitative Polymerase Chain Reaction (RT-qPCR)

The expression of genes associated with digestive enzymes [alpha 2A amylase (AMY2A), pancreatic lipase (PNLIP), cholecystokinin (CCK), and chymotrypsin-like elastase family member 1 (CELA1)], barrier functions and antimicrobial defense [beta-defensin 1 (DEFB1), fatty acid-binding protein-2 (FABP-2) and mucin-2 (MUC-2)], and proinflammatory cytokines [interleukin-1beta (IL-1β) and tumor necrosis factor-alpha (TNF-α)] was done. Total RNA was extracted via RNeasy Mini kits (Qiagen, Cat. No. 74104) following the kits’ instructions. RNA purity and concentration were determined using NanoDrop ND-8000 spectrophotometer (Thermo Fisher Scientific, Waltham, MA, USA). The cDNA was then synthesized using RevertAidTM H Minus kits (Fermentas Life Science, Pittsburgh, PA, USA) according to manufacturer’s instructions. The RT-qPCR assays were done using SsoAdvanced™ Universal SYBR Green^®^ Supermix (Bio-Rad, 1725274) according to the manufacturer’s instructions and analyzed using Stratagene™ MX3005P qPCR thermocycler (Agilent Technologies, Santa Clara, CA, USA). The sequences of primers of target genes are illustrated in Table 2. Target genes expression was normalized using glyceraldehyde 3-phosphate dehydrogenase (*GAPDH*) as a housekeeping gene. Relative fold changes in the target genes’ expression were determined using 2^−ΔΔCT^ method [48].

### 2.7. Campylobacter jejuni Challenge Model

A pandrug-resistant (PDR) and multi-virulent field *C. jejuni* strain was used in this experimental trial. It was previously recovered from cloacal swabs of recently slaughtered broiler chickens based on a previous research by one of the co-authors [51]. The strain was inoculated into Bolton broth (Oxoid, UK) for 48 h at 42 °C in microaerophilic conditions (10% CO_2_, 85% N_2_ and 5% O_2_) using an anaerobic jar (Sigma-Aldrich) with CampyGen sachets (Oxoid). The inoculated broth was then streaked onto modified charcoal cefoperazone deoxycholate agar (Oxoid, UK) plates that were incubated under the previous microaerophilic conditions. The strain was then evinced to be resistant to 24 antimicrobials (amoxicillin, ampicillin, sulbactam-ampicillin, amoxicillin-clavulanic acid, cephalothin, cefoxitin, cefoperazone, cefepime, imipenem, aztreonam, nalidixic acid, ciprofloxacin, trimethoprim-sulfamethoxazole, doxycycline, erythromycin, azithromycin, clarithromycin, tobramycin, gentamicin, amikacin, linezolid, chloramphenicol, colistin, and clindamycin) of 10 different antimicrobial categories being identified as PDR. The stain was also proved to harbor three substantial virulence factors, which have vital roles in its pathogenesis (*wlaN*, *virB11*, and *flaA*) as previously detailed) [52]. The challenge inoculum was adjusted to provide a viable concentration of 10^8^ CFU/mL [4]. At 30 days of age, the birds in all groups including control were orally infected through the crop gavage with 10^8^ CFU/mL of *C. jejuni* (1 mL per bird). The experimental infection was then affirmed following the appearance of clinical signs via re-isolation and identification of the infecting *C. jejuni* strain in addition to re-investigating its antimicrobial resistance pattern and virulence genes profiling.

### 2.8. Real-Time Quantitative Polymerase Chain Reaction (qPCR) for Evaluating C. jejuni Shedding and Colonization

Quantitative PCR assays were carried out to quantify the microbial populations including *Bifidobacterium*, *Enterobacteriaceae*, *Lactobacillus*, and *Clostridium* species in the avian intestinal contents at the end of the experimental period and to determine *C. jejuni* colonization in chickens’ cecal samples and its shedding in their fecal samples 3 and 7 days post-infection (33 and 35 d of age). Genome DNA was extracted with QIAamp DNA fast DNA stool kit (Qiagen, Hilden, Germany) adopting the guidance of the manufacturer. Purity and concentration of extracted DNA were determined using NanoDrop (Thermo Fisher Scientific). The sequences of the primers used for quantification of intestinal microbial populations and *C. jejuni* are illustrated in Table 2. The number of copies of DNA was determined, in triplicate, using Stratagene MX3005P^®^ RT-PCR instrument considering the created standard calibration curves prepared from serially diluted pure bacterial cultures and then the bacterial quantities were expressed as log^10^ of the CFU/g of the sample.

### 2.9. Statistical Analysis

The results were evaluated via one-way ANOVA test using SPSS Inc. program version 20 (IBM Corp., Armonk, New York, NY, USA). Differences among the results were expressed as the standard error of the mean (SEM) and variations between means were assessed at a probability level of 5% using Tukey’s test. All graphs were made using GraphPad Prism program Version 8 (San Diego, CA, USA).

## 3. Results

### 3.1. Efficacy of B. amyloliquefaciens-Loaded Nanoparticles on Growth Performance and Mortality Percentage of Broiler Chickens

The effect of adding BNPs in different doses to broilers’ diets within the whole experimental period (35 days) is shown in Table 1. Our data revealed that broiler chickens fed BNPs with higher doses (BNPs II and BNPs III) for 35 days exhibited significant (*p* ˂ 0.05) increases in their body weight gain (2499 and 2569 g/bird, respectively) compared to those fed BNPs I (2430 g/bird) and control diets free of BNPs (2297 g/bird). While there was a reduction in food intake in the groups of birds fed BNPs compared to those in the control group, their efficiency for food utilization was improved (*p* ˂ 0.05) specifically with higher BNPs doses as indicated by lower FCR in these groups compared to that in the control one. Notably, inclusion of BNPs with higher doses significantly (*p* ˂ 0.05) decreased mortality rates compared with the challenged group (Table 3).

### 3.2. Efficacy of B. amyloliquefaciens-Loaded Nanoparticles on Expression of Digestive Genes Controlling Digestion

**Figure 2:**
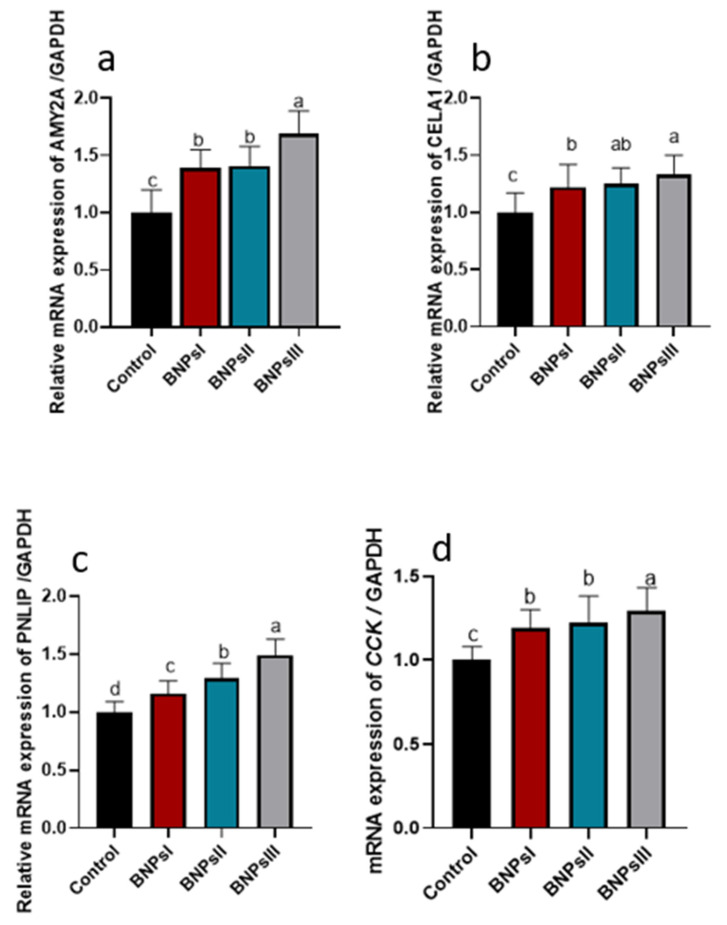
Effect of feeding Ross broilers (*n* = 5/group) different levels of *Bacillus amyloliquefaciens*-encapsulated nanoparticles (BNPs I, II, and III) on the expression of digestive enzymes-related genes compared to birds fed basal diet. Graphs represented the relative mRNA expression of alpha 2A amylase (*AMY2A*, (**a**)), chymotrypsin-like elastase family member 1 (*CELA1*, (**b**)), pancreatic lipase (*PNLIP*, (**c**)), and cholecystokinin (*CCK*, (**d**)). The data represented the relative mRNA expression normalized to *GAPDH* and the significance is *p* < 0.05. ^a–d^ Mean values within columns showing numerous letters differ statistically.

The analysis of genes expression associated with digestive enzymes including *AMY2a*, *PNLIP*, *CELA1,* and CCK is illustrated in Figure 2. The results indicated that the expression levels of *AMY2a* and *CCK* genes attended their peaks (*p* ˂ 0.05) following supplementation of BNPs III. A concentration-dependent manner upregulation of *PNLIP* gene (*p* ˂ 0.05) was detected following BNPs’ dilatory inclusion. Significant (*p* ˂ 0.05) upregulation of relative expression of *CELA1* gene was noticed in broilers fed diets supplemented with higher levels of BNPs compared to control ones fed diets free of BNPs.

### 3.3. Efficacy of B. amyloliquefaciens-Loaded Nanoparticles on Broiler Chickens’ Intestinal Microbial Populations

**Figure 3:**
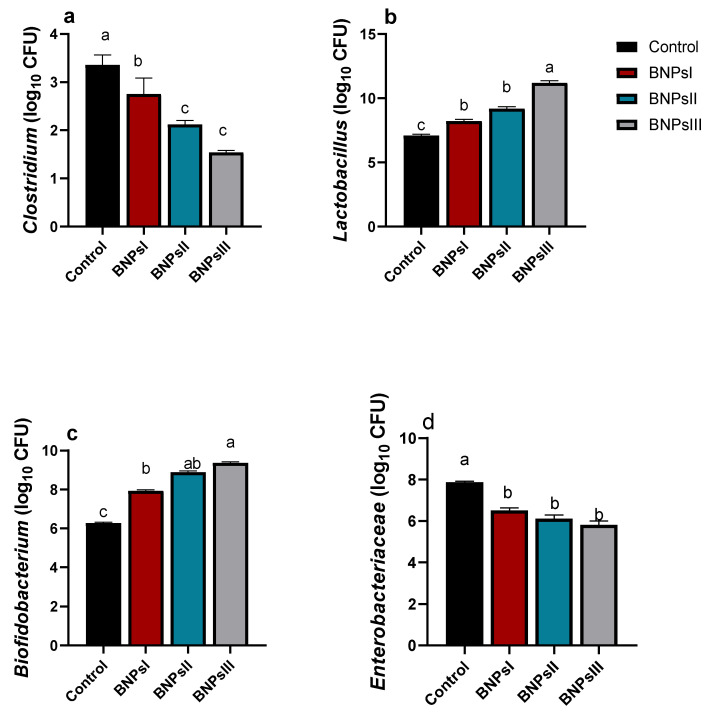
Effect of feeding Ross broilers (*n* = 5/group) different levels of *Bacillus amyloliquefaciens*-encapsulated nanoparticles (BNPs I, II, and III) on the intestinal microbial populations compared to birds fed basal diet. Graphs represented the counts of *Clostridium* (**a**), *Lactobacillus* (**b**), *Bifidobacterium* (**c**), and *Enterobacteriaceae* (**d**) expressed as log^10^ of the CFU/g of the sample and the significance is *p* < 0.05. ^a–d^ Mean values within columns showing numerous letters differ statistically.

The abundance of intestinal microbial populations following BNPs supplementation is illustrated in Figure 3. As expected, there were increases in the abundance of *Lactobacillus* and *Bifidobacterium* and reductions in *Clostridium* and *Enterobacteriaceae* as indicated by qPCR in groups of birds fed BNPs compared to control ones. Although the differences among the three groups of birds fed different doses of BNPs did not reach statistical significance in reduction of *Enterobacteriaceae* and *Clostridium* species abundance unlike the control group, PNPIII showed the highest tendency in their reduction. Conversely, a high tendency in increasing *Bifidobacterium* populations was noticed in BNPs-fed groups compared to the control one. Likewise, BNPs III-fed birds had significant (*p* ˂ 0.05) highest counts for both *Lactobacillus* and *Bifidobacterium* species.

### 3.4. Efficacy of B. amyloliquefaciens-Loaded Nanoparticles on Intestinal Barrier and Cytokines-Associated Genes 

**Figure 4:**
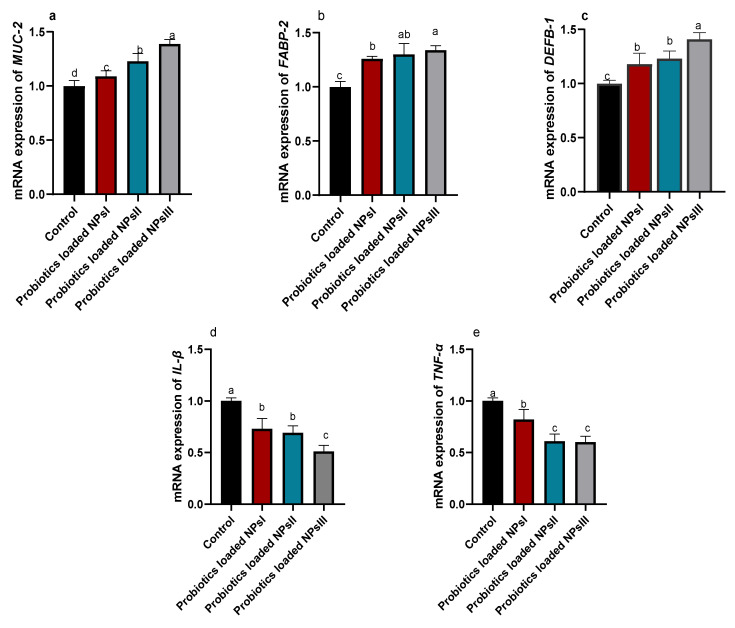
Effect of feeding Ross broilers (*n* = 5/group) different levels of *Bacillus amyloliquefaciens*-encapsulated nanoparticles (BNPs I, II, and III) on the relative mRNA expression of mucin-2 (*MUC-2*, (**a**)), fatty acid-binding protein-2 (*FABP-2*, (**b**)), beta-defensin 1 (*DEFB-1*, (**c**)), interleukin-1beta (*IL-1β*, (**d**)), and tumor necrosis factor-alpha (*TNF-α*, (**e**)). The data represented the relative mRNA expression normalized to *GAPDH* and the significance is *p* < 0.05. ^a–d^ Mean values within columns showing numerous letters differ statistically.

Analysis of the relative mRNA expression of genes related to intestinal barrier and antimicrobial defense including *DEFB1*, *FABP-2* and *MUC-2,* and proinflammatory cytokines comprising *IL-β* and *TNF-α* is depicted in Figure 4. We observed an overwhelming increase in the expression of *MUC-2* gene after supplementing BNPs in broiler chickens’ diets in a dose-reliant way. In addition, a proportional (*p* ˂ 0.05) increase in the expression of *FABP-2* gene was demonstrated in broiler chickens with increasing the dose of dietary BNPs when compared with the control group. The expression of *DEFB1* gene achieved its highest peak in BNPs III-fed birds. Notably, prominent downregulation (*p* < 0.05) was noticed regarding *IL-1β* and *TNF-α* genes, especially after feeding higher BNPs levels when compared to the control group.

### 3.5. Efficacy of B. amyloliquefaciens-Loaded Nanoparticles on Shedding and Colonization of C. jejuni 

**Figure 5:**
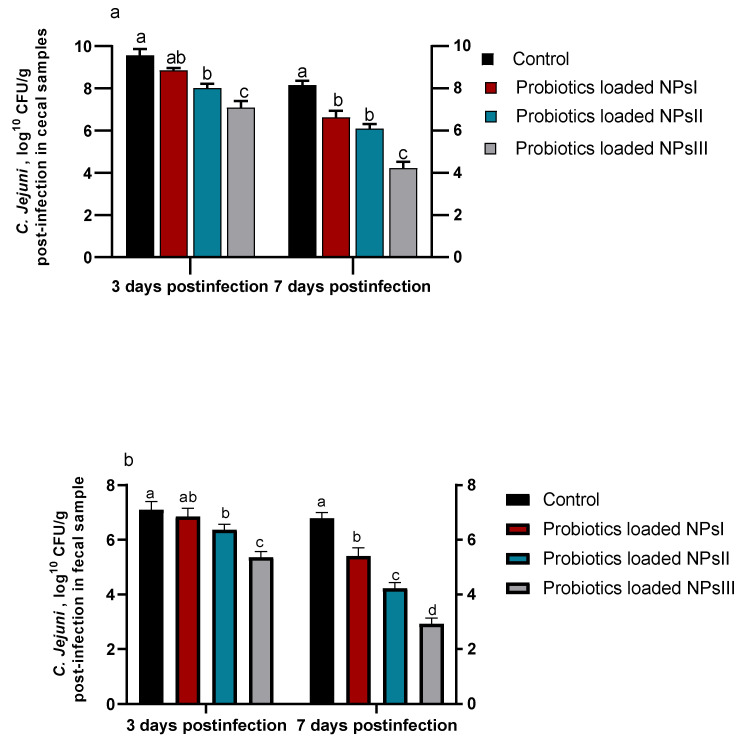
Effect of feeding Ross broilers (*n* = 5/group) different levels of *Bacillus amyloliquefaciens*-encapsulated nanoparticles (BNPs I, II, and III) on colonization and shedding of *Campylobacter jejuni* (*C. jejuni*) in cecal (**a**) and fecal (**b**) samples at 3 and 7 days post-infection compared to birds fed basal diet and the significance is *p* < 0.05. ^a–d^ Mean values within columns showing numerous letters differ statistically.

Investigating *C. jejuni* shedding and colonization in fecal and cecal samples 3 and 7 days post-infection using qPCR assay is shown in Figure 5. After 3 and 7 days of *C. jejuni* infection, there was a tendency of fecal and cecal *C. jejuni* counts toward reduction within groups of birds fed BNPs with different doses (BNPs I, BNPs II, and BNPs III) compared to those in the control group fed diets free of BNPs. Notably, a lower trend of *C. jejuni* counts was detected in fecal samples than cecal ones 3 and 7 days post-infection. At both intervals, inclusion of BNPs III in birds’ diets remarkably (*p* ˂ 0.05) reduced *C. jejuni* populations considering both sample types unlike the control group. Seven days post-infection, *C. jejuni* counts were significantly (*p* ˂ 0.05) reduced in fecal and cecal samples of birds fed BNPs III compared to those in the control group (2.93 and 4.22 CFU/g vs. 6.79 and 8.16 CFU/g), respectively.

## 4. Discussion

Higher productivity in the poultry industry has been complemented by the balance between nutrition, intestinal health, and animal welfare. Additionally, there are many impacts threatening this productivity such as the emergence of a large assortment of pathogens and bacterial resistance [53]. The concept of using in-feed live probiotics strains in the poultry industry not only exerts a beneficial effect on overall growth performance parameters [54], but also signifies their use in reducing and eliminating the colonization of pathogenic bacteria, such as *C. jejuni* [19]. Nevertheless, gut colonization and effectiveness of supplemented probiotics depend on various aspects involving the specificity of the strains relative to the host, digestive enzymes, bile acids, availability, nutritional status of the host, intestinal pH, stress, and form of substrate (prebiotics) [55]. Thus, offering probiotic living cells with a physical barrier for escaping from unfavorable environmental conditions is a crucial consequence, which currently has obtained substantial interest [56]. In this study, encapsulation of *B. amyloliquefaciens* in chitosan nanoparticles had beneficial effects on broiler chicken growth performance and reduced the colonization and shedding of *C. jejuni*.

*C. jejuni* represents the most prevalent species accountable for 84% of cases diagnosed recently in Europe [57]. Poultry is the main carrier of the bacteria and is responsible for the most outbreaks of campylobacteriosis through consumption of contaminated poultry meat [43]. The bacteria can colonize and persist in the chicken gut during their lifetime without causing diseases [31]. The emergence of MDR, extensively drug-resistant, and PDR strains [58,59], especially *C. jejuni*, which is resistant to the main drugs used in the treatment of severe post-acute sequelae of campylobacteriosis (fluoroquinolone and macrolides) evoked a warning sign about hazardous uses of antibiotics for enhancing poultry growth performance and treatment of bacterial infections [60]. The use of probiotics such as *Lactobacillus*, *Bacillus*, and *Bifidobacterium* has been shown to be a promising alternative solution for reduction of pathogen burden in the avian gut and consequently breaks the transmission cycle to humans through the food chain. However, the effects of inclusion of BNPs in poultry diets to decrease the [60] colonization and shedding of *C. jejuni* need to be defined.

In this study, we have shown that supplementation of *B. amyloliquefaciens* coated within chitosan nanoparticles, especially at high doses in broilers’ diets for 35 days, promoted their growth performance and increased their body weight gain. Moreover, feed utilization efficiency of broilers was improved as indicated by significant (*p* < 0.05) reduction in FCR and increase in BWG, especially in BNPs III-fed group. These results are consistent with previous data regarding the improvement in growth performance of broilers fed *B. licheniformis* spores supplement either alone or combined with mannan oligosaccharide feed additives [61]. Correspondingly, probiotics can modify the intestinal ecosystem by supplying digestion enzymes, reducing pH, and increasing the activity of enzymes in the gastrointestinal tract [62,63]. Similarly, [64] reported a significant improvement in FCR and growth performance of broilers fed dried brewer grain (10%) subjected to probiotics fermentation. Different formulations used for probiotics delivery would affect their absorption; therefore, in this study, chitosan nanoencapsulated probiotics improved their resistance against gastric and bile acids, which consequently increased their bioavailability, absorption, and sustained effectiveness during the overall period of experiments (35 days). The growth performance of birds was strongly correlated with the digestibility and utilization efficiency of feed [16]. Moreover, it has been known that dietary composition of broilers’ rations could modulate the expression of digestive enzymes and nutrient transport-related genes [57,65]. Therefore, our study revealed that a group of birds fed BNPs III for 35 days exhibited significant increase in relative mRNA expression of pancreatic digestive enzyme-linked genes, such as *AMY2A, PNLIP, CELA1,* and *CCK*. Aligned with our data, [64] stated that feeding microbially fermented dried brewer grains not only improved growth performance and carcass dressing, but also stimulated expression of pancreatic digestive enzymes encoding genes as *AMY2A, PNLIP, CELA1,* and *CCK*.

Microbiota and their metabolites prompt gut enteroendocrine cells to secrete gut hormones, which consequently influence metabolism [66]. Our results indicated the ability of BNPs to stimulate gut hormones, such as CCK, which plays important roles in gastric motility, appetite, bile acids, and pancreatic enzymes release [66,67]. Although less is known about how probiotics regulate CCK secretion, a recent research showed that feeding broilers for 42 days on microbially fermented olive pomace via two stages of solid fermentation using *Bacillus subtilis* followed by *Lactobacillus casei* upregulated the genes encoding pancreatic enzymes: *CCK*, *AMY2A, PNLIP,* and *CELA1* [18]. Furthermore, dietary inclusion of microbially fermented soybean meal enhanced pancreatic enzymes’ activities in broilers [68]. Moreover, dietary supplementation of *B. amyloliquefaciens* had significant efficacy in enhancing chymotrypsin, amylase, and lipase activities compared to groups fed antibiotics alone [69]. Similarly, it has been reported that *B. amyloliquefaciens* (2 × 10^5^ CFU/g diet) pretreatment either alone or combined with mannan oligosaccharides improved metabolic activity of intestinal cell energy-related genes through upregulation of mRNA genes expression of enzymes implicated in protein digestion. The mechanism by which probiotics regulate digestive enzymes secretion needs to be deciphered. However, this may be attributed to the role of probiotics in restoring intestinal normal architecture and achievement of larger healthy surface area for nutrient assimilation [70]. Moreover, the role of probiotics in increasing the relative mRNA expression of pancreatic digestive enzymes could be related to their vital role in the activity of enteroendocrine cells to express the pancreatic enzymes encoding genes. Additionally, increasing the expression of genes encoding pancreatic lipase associated enzymes (PNLIP and CCK) could be secondary to the production of probiotics’ metabolites including short chain fatty acids [71].

Our data indicated increases in the abundance of *Bifidobacterium* and *Lactobacillus* species, which was accompanied by reduction in *Enterobacteriaceae* and *Clostridium* species populations. In parallel, probiotics not only exert their beneficial effects on host resistance through improving its immunity, epithelial function, and competitive exclusion of pathogens burden within the avian gut, but also through their effects on rescuing the normal microbial populations of the intestine via favoring the growth of beneficial commensals and inhibiting that of the opportunistic ones [72]. Moreover, Bao et al. [73]. reported an increase in the abundance of *Lactobacillus* species combined with reduction in *Enterobacteriaceae* and *Clostridiales* counts after inclusion of *B. amyloliquefaciens* in poultry diet compared to poultry fed a basal diet [73]. The increase of *Lactobacillus* and *Bifidobacterium* within the cecal contents of broilers may be attributed to the ability of BNPs to produce extracellular enzymes as phytase, amylase, xylanase, and β-glucanase, which could improve food digestibility and utilization [74]. In addition, accumulation of short chain fatty acids produced within microbial metabolic processes confers a suitable pH that would support the growth of beneficial bacteria and prevent that of harmful ones [73,74]. Both *Lactobacillus* and *Bifidobacterium* provide substrates for augmenting the growth of butyrate-producing bacteria, which stimulates epithelial regeneration and improves host energy metabolism [61].

Improvement of epithelial barrier functions as indicated by upregulation of *MUC2* and *IgA* genes expression was observed following administration of *Bacillus coagulans* to chickens [75]. Consistent with this finding, we found that improvement of growth performance of birds was correlated with upregulation of epithelial barrier functions-related genes including *MUC2*, *DEFB1*, and *FABP2*, specifically, in birds supplemented with BNPs III in their diet for 35 days. Avian defensin represents the innate antimicrobial peptide with a wide range of spectrum against bacteria, fungi, and protozoa [76,77]. Moreover, its ability to kill bacteria is through alteration in cell membrane permeability and immunomodulatory functions involving chemoattraction for leukocytes to the injury site and elimination of bacterial lipopolysaccharides and lipoteichoic acids triggering proinflammatory response [76]. The upregulation of avian defensin following BNPs supplementation suggests its role as an antimicrobial peptide against MDR bacteria as proved previously [78]. Mucus is the first physical barrier protecting epithelial cells from microbial translocation and inflammatory response [79]. Likewise, intestinal barrier is strengthened by a glycosylated mucin-rich layer produced by goblet cells [80]. Supporting this view, probiotics have been proven to strengthen the integrity of the intestinal barrier by elevating the number of goblet cells that support the mucus layer [81]. A previous study described that many *Bacillus* species were evidenced to upregulate the expression of intestinal mucin [82] as demonstrated in our study. *FABP-2* gene expression has been indicated as a biomarker for intestinal barrier in broilers’ gut and its higher expression signifies its superior role in intestinal function [83]. Similarly, our data showed upregulation of *FABP-2* relative mRNA expression, which suggested that BNPs enhanced epithelial integrity and functions. In the present study, downregulation of intestinal gene expression levels of proinflammatory cytokines (*TNF-α* and *IL-1β*) were observed in broilers fed higher levels of BNPs. This observation indicated their suppressive role on proinflammatory cytokines, which in turn counteracted the inflammation [80,84,85].

Several studies evaluated the *in vitro* and *in vivo* anti-campylobacter activities of different probiotics strains [15,19]. However, the effect of dietary inclusion of probiotics-loaded nanocarriers on the improvement of birds’ resistance against *C. jejuni* and their interaction with host epithelial cells need to be explicitly defined. Our data further demonstrated that the aforementioned beneficial effects of BNPs, especially with higher doses, led to significant reduction in cecal colonization and fecal shedding of *C. jejuni*. These data suggested the ability of BNPs to competitively exclude *C. jejuni* from broilers’ intestine and prevent its colonization. From what has been declared above, our data explained several possible mechanisms by which BNPs could mediate the exclusion and inhibition of *C. jejuni* colonization and translocation within the avian gut. These mechanisms are mediated firstly through the improvement of nutrient digestibility and assimilation and secondly via their effects on improving intestinal morphology and functions through upregulation of *FABP2* and *MUC2* genes, which increased mucus secretion and prevented bacterial adhesion. Lastly, they exert antimicrobial impacts either through secretion of antimicrobial peptides, such as β-defensin, that breaks down the bacterial cell wall and consequently activates the innate immune response to control inflammatory process and prevent bacterial translocation. 

## 5. Conclusions

Our data suggested that encapsulation of *B. amyloliquefaciens* as a probiotic bacterium by chitosan nanoparticles enhanced its bioavailability and maintained its beneficial effects in the broiler chicken’s gut. The augmented growth promoter properties of BNPs were detected in our study and supported by higher expression of digestive enzymes-related genes. Friendly gut bacterial species including *Bifidobacterium* and *Lactobacillus* species outnumbered the unfriendly ones following inclusion of dietary BNPs. Supplementation of BNPs could also enhanced gut barrier functions and consequently decrease *C. jejuni* colonization with a superior outcome of minimizing its fecal shedding.

## Figures and Tables

**Table 1:** The ingredients of basal diets and nutrient composition.

Item	Starter (Days 1–10)	Grower (Days 11–20)	Finisher (Days 21–35)
Ingredient (%) Soybean meal (48)	34.4	30.8	25.8
Ground yellow corn	59	61.5	65.5
Soybean oil	1.8	3	4
Calcium carbonate	1.2	1.2	1.2
Dicalcium phosphate	1.5	1.5	1.5
Common salt	0.3	0.3	0.3
Choline chloride	0.2	0.2	0.2
Anti-mycotoxin	0.1	0.1	0.1
L-Lysine HCL (Lysin, 78%)	0.35	0.3	0.3
DL-Methionine (Methionine, 99%)	0.25	0.2	0.2
Premix *	0.3	0.3	0.3
Chemical composition			
Lysine (%)	1.5	1.3	1.2
Methionine (%)	0.6	0.5	0.5
Available phosphorus (%)	0.5	0.5	0.5
Calcium (%)	1.2	1.2	1.2
Ether extract (%)	4.3	5.6	6.6
Crude fiber (%)	2.6	2.6	2.5
Crude protein (%)	23	21.5	19.5
Metabolizable energy (kcal/kg)	3106	3103	3200

* Premix provided per kilogram of diet: Se (selenate), 0.3 mg; I (iodide), 1.3 mg; Mn (sulphate and oxide), 100 mg; Fe (sulphate), 30 mg; Cu (sulphate), 14 mg; Zn (sulphate and oxide), 120 mg; thiamine, 4 mg; pyridoxine, 6 mg; pantothenate, 14 mg; niacin, 50 mg; biotin, 200 μg; cyanocobalamin, 15 μg; riboflavin, 7 mg; cholecalciferol, 6000 IU; retinol, 10.000 IU; folate, 3 mg; and tocopherol acetate, 70 mg.

**Table 2:** Target genes and primers used for qPCR assays.

Specificity/Target Gene	Primer Sequence (5′–3′)	Accession No./ Reference
Digestive enzymes		
*AMY2A*	F: CGGAGTGGATGTTAACGACTGG R: ATGTTCGCAGACCCAGTCATTG	NM_001001473.2
*CELA1*	F-AGCGTAAGGAAATGGGGTGG R-GTGGAGACCCCATGCAAGTC	XM_015300368.1
*CCK*	F: AGGTTCCACTGGGAGGTTCT R: CGCCTGCTGTTCTTTAGGAG	XM_015281332.1
*PNLIP*	F: GCATCTGGGAAG↓GAACTAGGG R: TGAACCACAAGCATAGCCCA	NM_001277382.1
Intestinal microbiota/*16S rRNA*		
*Lactobacillus* species	F: CACCGCTACACATGGAG R: AGCAGTAGGGAATCTTCCA	[49]
*Bifidobacterium* species	F-GCGTCCGCTGTGGGC R-CTTCTCCGGCATGGTGTTG	[49]
*Enterobacteriaceae*	F: CATTGACGTTACCCGCAGAAGAAGC R: CTCTACGAGACTCAAGCTTGC	[49]
*Clostridium* species	F: AAAGGAAGATTAATACCGCATAA R: ATCTTGCGACCGTACTCCCC	[50]
Barrier functions and antimicrobial defense		
*MUC-2*	F-AAACAACGGCCATGTTTCAT R-GTGTGACACTGGTGTGCTGA	NM_001318434
*FABP-2*	F: AGGCTCTTGGAACCTGGAAG R: CTTGGCTTCAACTCCTTCGT	NM_001007923
*DEFB1*	F: AGCCTGTCTGCCTGGAGTAG R: GATGAGGAGAGGCTTCATGG	XM017337690.1
Proinflammatory cytokines		
*IL-1β*	F: GCTCTACATGTCGTGTGTGATGAG R: TGTCGATGTCCCGCATGA	NM_204524
*TNF-α*	F: CGTTTGGGAGTGGGCTTTAA R: GCTGATGGCAGAGGCAGAA	NM_204267.1
*Campylobacter jejuni*		
*mapA*	F: CTATTTTATTTTTGAGTGCTTGTG R: GCTTTATTTGCCATTTGTTTTATTA	[4]
Internal control		
*GAPDH*	F: GGTGGTGCTAAGCGTGTTA R: CCCTCCACAATGCCAA	X01578.1

*AMY2A*: alpha 2A amylase, *CELA1*: chymotrypsin-like elastase family member 1, *CCK*: cholecystokinin, *PNLIP*: pancreatic lipase, *MUC*-2: mucin-2, *FABP*-2: fatty acid-binding protein-2, *DEFB1*: beta-defensin 1, *IL-1β*: interleukin-1beta, *TNF-α*: tumor necrosis factor-alpha, *GAPDH*: glyceraldehyde 3-phosphate dehydrogenase.

**Table 3:** Effect of feeding male Ross broilers a diet formulated with three different levels of *Bacillus amyloliquefaciens*-encapsulated nanoparticles (BNPs I, II, III) on growth performance parameters.

Parameter	Control	BNPs I	BNPs II	BNPs III	*p* Value	SEM
Initial body weight (g/bird)	42.00	41.40	42.60	42.60	0.99	0.71
Overall growth						
Final body weight, g/bird	2339 ^c^	2471 ^b^	2542 ^a^	2612 ^a^	<0.001	21.54
Body weight gain, g/bird	2297 ^c^	2430 ^b^	2499 ^a^	2569 ^a^	<0.001	18.63
Feed intake, g/bird	4163 ^a^	3925 ^b^	3740 ^c^	3800 ^bc^	<0.001	29.78
Feed conversion ratio	1.82 ^a^	1.61 ^b^	1.50 ^bc^	1.48 ^c^	<0.001	0.05
Mortality % (1–35 d)	3 ^a^	2 ^ab^	1 ^b^	1 ^b^	0.025	0.37

Control: group of birds fed basal diet; BNPs I: group of birds fed diet containing *Bacillus amyloliquefaciens*-encapsulated nanoparticles at the level of 2.5 × 10^5^ CFU/gm; BNPs II: group of birds fed diet containing *Bacillus amyloliquefaciens*-encapsulated nanoparticles at the level of 5 × 10^5^ CFU/gm; BNPs III: group of birds fed diet containing *Bacillus amyloliquefaciens*-encapsulated nanoparticles at the level of 7.5 × 10^5^ CFU/gm; SEM: standard error of the mean. Values with various letters in the same row differ significantly (*p* < 0.05).

## Data Availability

Data presented in this research are offered upon request from the corresponding author.
